# BioTile, A Perl based tool for the identification of differentially enriched regions in tiling microarray data

**DOI:** 10.1186/1471-2105-14-76

**Published:** 2013-03-03

**Authors:** Jerry Guintivano, Michal Arad, Kellie LK Tamashiro, Todd D Gould, Zachary A Kaminsky

**Affiliations:** 1 Mood Disorders Center, Department of Psychiatry and Behavioral Sciences, Johns Hopkins University School of Medicine, Baltimore, USA; 2 Department of Psychiatry, University of Maryland School of Medicine, Baltimore, USA; 3 Department of Pharmacology, University of Maryland School of Medicine, Baltimore, USA; 4 Department of Anatomy & Neurobiology, University of Maryland School of Medicine, Baltimore, USA

**Keywords:** DNA methylation, Differentially methylated region, Tiling microarray, Algorithm, Epigenetic

## Abstract

**Background:**

Genome-wide tiling array experiments are increasingly used for the analysis of DNA methylation. Because DNA methylation patterns are tissue and cell type specific, the detection of differentially methylated regions (DMRs) with small effect size is a necessary feature of tiling microarray ‘peak’ finding algorithms, as cellular heterogeneity within a studied tissue may lead to a dilution of the phenotypically relevant effects. Additionally, the ability to detect short length DMRs is necessary as biologically relevant signal may occur in focused regions throughout the genome.

**Results:**

We present a free open-source Perl application, Binding Intensity Only Tile array analysis or “BioTile”, for the identification of differentially enriched regions (DERs) in tiling array data. The application of BioTile to non-smoothed data allows for the identification of shorter length and smaller effect-size DERs, while correcting for probe specific variation by inversely weighting on probe variance through a permutation corrected meta-analysis procedure employed at identified regions. BioTile exhibits higher power to identify significant DERs of low effect size and across shorter genomic stretches as compared to other peak finding algorithms, while not sacrificing power to detect longer DERs.

**Conclusion:**

BioTile represents an easy to use analysis option applicable to multiple microarray platforms, allowing for its integration into the analysis workflow of array data analysis.

## Background

Genome-wide DNA methylation studies are becoming increasingly used in search of etiological factors contributing to complex non-Mendelian disease, as the susceptibility of DNA methylation to environmental influences and its potential for metastable drift may account for complex disease features, such as a discordance of monozygotic twins, parent of origin effects, an unequal frequency of affected males and females, complex inheritance, and a late age at onset, among others [1-3]. DNA methylation changes in the brain are becoming increasingly recognized as important mediators of behavioral phenotypes in model organisms and psychiatric disease in humans [4-7].

Despite the likelihood of epigenetic changes as etiological factors contributing to psychiatric disease risk, the success of the first round of epigenomic studies has been limited [8]. In the first epigenomic profiling studies performed in major psychosis, Mill et al. found moderate fold changes in prefrontal cortex DNA methylation. In the *WDR18* glutamate receptor subunit gene, an 8% DNA methylation difference was detected between males with schizophrenia and controls, while female patients with bipolar disorder were 6% more methylated than controls at the *RPL39* gene [9]. No significant differences were found in an analysis of 50 loci in temporal cortex of schizophrenia affected individuals [10]. A recent methylome profiling study in major depressive disorder (MDD) did not identify any significant loci after correction for multiple testing; however, they did successfully validated a 60% of the top nominally significant differences [11]. Of these, the largest depression associated effect size was 22%.

A consistent feature of these studies is the low effect size associations detected in the brain. A probable explanation for these observations is that true disease differences exist in a subpopulation of cells that are subject to dilution by disease non-relevant cell types, a factor particularly relevant in the brain, which represents one of the most cellularly heterogeneous organs in the body. This situation calls for algorithms capable of detecting DMRs of small effect size in order to direct downstream validation and follow up functional studies, such as cell type specific analyses. In this regard, the ability of a DMR detection technique to adjust for covariates such as cellular heterogeneity, medication status, or age are of particular interest in psychiatric phenotypes but to date, few available algorithms for DMR detection allow for these adjustments.

Another factor that remains at issue is that phenotypically relevant epigenetic changes may occur over relatively small regions. A number of locus specific studies highlight the importance of short genomic regions in regulating phenotypic outcome. Epigenetic changes spanning short genomic regions have been identified in imprinting control regions, over exonic regions that may direct alternative splicing, and at transcription factor binding sites that have been associated with early life trauma exposure or major psychosis [9,12-14]. The power to identify short DMRs is an important facet of DMR finding algorithms used in studies searching for small epigenetic aberrations conferring phenotypic variation.

The application of tiling array technology to the study of DNA methylation has greatly increased the resolution over earlier microarray based technologies and added to the potential to discover novel epigenetic changes. Tiling array experiments are based on measuring the genomic locations of enriched DNA fragments that hybridize across adjacently located probes called tiles. The experiments performed prior to hybridization involve enriching for the molecular marker of interest, either through antibody based immunoprecipitation employed in ChIP-chip [15], MeDIP [16,17], or through enzymatically selecting a portion of the genome, such as with methylation sensitive restriction enzymes as is employed in numerous DNA methylome techniques [18-21]. The enriched fractions are fragmented to improve target specificity, generally to lengths of 50–200 base pairs. After microarray hybridization, the combinatorial effects of fragment binding to specific genomic locations will result in peaks of signal intensity after data processing that may be detected by downstream data analysis applications.

A number of excellent programs that contain peak finding algorithms are available for the analysis of tiling array data, some of which include Ringo [22], ChiPOTle [23], CHARM [19], TileMap [24], ACME [25], and MPEAK [26], among others. There is a large degree of variation in the statistical methods employed, the ease of use, and the versatility across multiple experiment types. For example, many of these algorithms, such as CHARM and Ringo, were designed for one type of platform, such as NimbleGen arrays, but can now be applied to other datasets. Others, such as ChiPOTle are limited in the number of probes that can be analyzed (IE: 60,000), which makes it difficult to apply to larger tiling array datasets. With the exception of CHARM, these DMR finding algorithms are confined to the investigation of group classifiers as opposed to quantitative variables such as multiple treatment doses or age and do not allow for the correction of covariates prior to peak identification and statistical evaluation.

Cumulatively, the application of tiling array analyses to DNA methylation in heterogeneous tissues, such as brain, require the ability to detect DMRs of small effect size and of short length. A simple analysis paradigm applicable to multiple microarray platforms and satisfying these requirements will add to the successful identification of phenotypically relevant epigenetic variation across a diverse range of phenotypes. To address these issues we present an open source, freely available Perl application referred to as “Binding Intensity Only Tiling array analysis” or “BioTile”. The BioTile algorithm is ideally suited to the identification of small length and low effect size DMRs, while not sacrificing power to detect longer DMRs, and is applicable across a range of tiling microarray platforms.

## Implementation

BioTile is a single software application written in the Perl programming language designed for the identification of differentially enriched regions (DERs) in tiling microarray datasets (Additional file 1, Additional file 2, Additional file 3, Additional file 4). BioTile identifies DERs associated with either a group classifier, such as case vs. control, or continuous variables. To achieve this, BioTile first calculates the slope of a linear model between dependent and independent variables for each probe individually. Subsequently, stretches of adjacent probes exhibiting slope values above zero or below zero for greater than three probes are identified as potential DERs and are subsequently subjected to statistical evaluation. If specified by the user, BioTile will return DERs associated with the outcome of an additive multiple linear regression model, enabling control of covariates that may be present in psychiatric studies such as smoking status, medication history, age at onset, and cellular heterogeneity, among others. The default threshold of three probes was selected to maximize the resolution of the technique and therefore the ability of the algorithm to identify small DERs. This probe length corresponds to the average fragment length generated in most experimental enrichment steps and the probe spacing per array platform (3 probes: Affymetrix ~ 105 bp, CHARM ~ 100 bp, Agilent ~ 100 bp); however, this threshold can be defined by the user. The distributions of DNA methylation across each probe in an implicated DER are subsequently evaluated statistically using a permutation corrected meta-analysis. The linear regression slope and squared standard error of the slope per probe in an implicated DER are used to calculate Cochran’s meta-statistic, Q, according to the following formula:

$$\text{Q}=\sum_{j=1}^{n}\left[{\frac{\left(m_{j}-\left[\frac{\sum_{i=1}^{n}\left(\frac{1}{{\text{SE}}_{m_{i}}^{2}}*\;m_{i}\right)}{\sum_{i=1}^{\text{n}}\frac{1}{{\text{SE}}_{m_{i}}^{2}}}\right]\right)}{{\text{SE}}_{m_{j}}^{2}}}^{2}\right]$$

where m represents the slope per probe and SE^2^_m_ represents the squared standard error of the slope calculated first at each i^th^ probe then each j^th^ probe in a DER consisting of n probes [27]. A permutation step is implemented to control for correlation among neighboring array tiles such that diagnostic criteria are shuffled at random for a number of iterations specified by the user (default 1000) and a null distribution of meta-statistics is generated. Significance is determined by calculating the quantile of the original meta-statistic, Q, relative to the null distribution for each DER. The output of the algorithm is a list of genomic regions (DER start and stop coordinates) differentially enriched between groups, each with its corresponding mean and maximum microarray fold difference and p value that can subsequently be corrected for multiple testing. The original meta-analysis Q statistic is also supplied to enable ranking of DERs that were returned with the same p-value such that a higher Q is indicative of a higher significance.

The software tool is designed for processing and DER peak finding in normalized datasets and is meant for use following standard quality assessment and data normalization steps. Resultantly, it is compatible with all single and dual channel microarray platforms. Use of the algorithm requires only a formatted data matrix containing chromosomal coordinates as well as an annotation file denoting the comparison of interest and containing relevant covariates.

## Results and discussion

The goal of any DER peak finding algorithm should be to maximize the probability that identified DERs represent regions of true biological variation between groups and are not the result of random technical variation within the experimental system. Generally speaking, biologically relevant regions will have a higher percentage of individually significant probe signals; as such regions are more likely to result in enrichment of DNA fragments likely to hybridize to a given area. However, due to the combinatorial nature of fragments hybridizing to a series of adjacent tiles, signals will be stronger at some probes and not others, resulting in a peak in which not all individual probes are significant. Conversely, technical variation in genome-scale experiments will generally appear to be chance occurrences of single probes appearing statistically significant between groups.

In order to minimize the identification of false positives and maximize DER peak identification, BioTile employs the use of a permutation corrected meta-analysis step capable of detecting peaks comprised of as few as three adjacent probes. As BioTile identifies DERs by identifying regions where the pair wise difference between the two groups is consistently above or below zero for a stretch longer than 3 probes, if false positive signals are located within regions where background levels are in the same direction, they will be included in the list of DERs to evaluate statistically. The implementation of the meta-analytical step as compared to more traditional statistics is more robust to these situations and can lead to the identification of DERs comprised of a higher percentage of individually significant probe values, non-biased by random probes of high difference. This is because the degree to which each probe contributes to the cumulative meta-statistic of a perspective DER will be inversely weighted by its variance, and the inclusion of noisy background probe levels will reduce the meta-statistic and thus significance of spuriously generated DERs.

**Figure 1:**
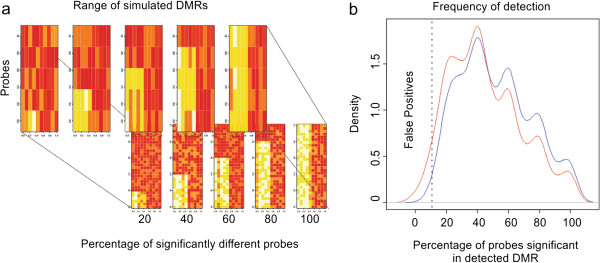
**Meta-analysis vs. t statistic performance. a**.) Heat maps of randomly generated DMRs among 5 cases and 5 controls where 20,40,60,80, and 100% of probes are significant. The foreground and background panes depict DMRs spanning 5 and 30 probes, respectively. **b**.) Density plots of the percentage of significant probes within DMRs detected by BioTile (blue) and the average *t*-test method (red).

To model this scenario, we generated a simulation DNA methylation dataset across a series of randomly permuted DMRs of variable probe lengths, ranging from 5 to 30, (~175 bp to ~1 kb based on Affymetrix probe spacing), with N = 5 cases, N = 5 controls, and a ~ 10 ± 0.1% DNA methylation difference on average between groups. The percentage of probes significantly different between cases and controls varied by 20%, 40%, 60%, 80%, and 100% per DMR (Figure 1a). For each scenario, 100 permutations of randomly generated DMRs were created to match the above criteria. We then compared the performance of a probe-wise permuted meta-analysis to a student’s *t*-test of the average DNA methylation value across probes in a simulated DMR. DMRs found significant below p = 0.05 by the meta-analytical technique showed a higher percentage of individual probe positions demonstrating significance (Mean Percent Detected BioTile = 0.51 ± 0.00016, Mean Percent Detected *T*-test = 0.45 ± 0.00011, p = 5.4 × 10^-6^)(Figure 1b). The frequency of false positive DMRs (those with less than 1% of probes being different) detected below the 5% p-value threshold was significantly lower in the meta-analytical method as compared to the *t*-test method (Fisher’s Exact OR = 0.18, p = 2.67 × 10^-12^). These results highlight that the meta-analytical approach is more robust to the influence of statistical outliers and is better suited to identifying DMRs with a high probability of independent validation.

### Comparison to available peak finding algorithms

#### Data simulation

**Figure 2:**
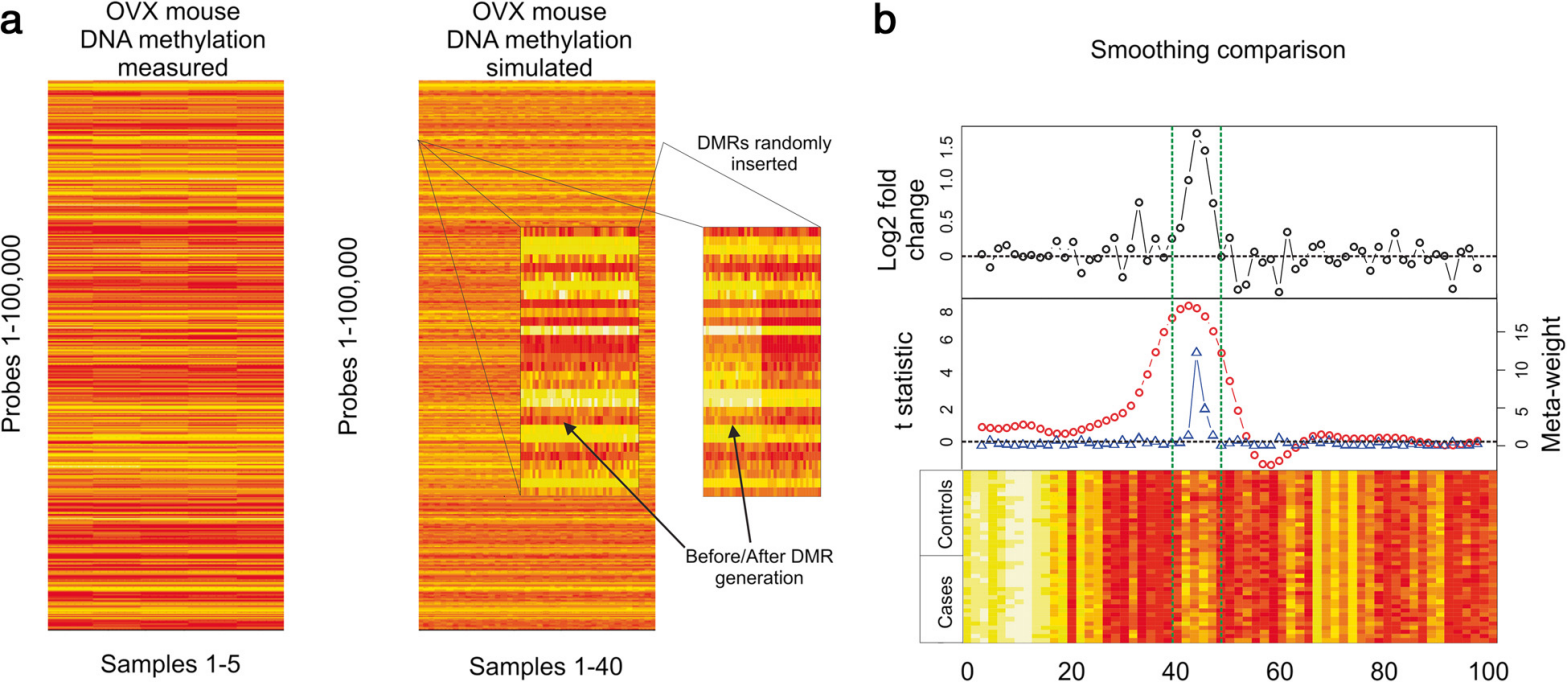
**Data simulation and smoothing performance. a**.) Heat maps of 100,000 probes derived from DNA methylation profiling of 5 control hippocampal samples from OVX C57BL/6 J mice and a simulated data matrix where probe mean and probe variance were modeled on the empirical dataset. An example of a simulated DMR inserted data matrix is depicted. **b**.) A plot of an example DMR 5 probes long exhibiting an average log_2_ fold change of 1. Mean group-wise differences are plotted in black (y-axis, top) as a function of the relative probe position (x-axis, top). The smoothed t-statistics (left y-axis, middle, red) generated by the CHARM algorithm and the relative meta-analysis weights generated by BioTile (right y-axis, middle, blue), are plotted. A heat map of the location is depicted (bottom). Vertical green lines denote the location of the inserted DMR.

To test the BioTile algorithm’s capability of identifying DMRs located within an actual tiling array dataset, we generated a simulated Affymetrix tiling array experiment according to the following strategy. Five female C57BL/6 J mice underwent ovariectomy at eight weeks of age followed by sacrifice 28 days later. Hippocampal tissue was isolated through cryostat sectioning and micro punch followed by genomic DNA isolation. Hippocampal DNA methylation profiles were generated using *HpaII* and *HinP1I* methylation sensitive restriction enzyme based enrichment strategy according to previously established protocols on Affymetrix Mouse Promoter 1.0R Tiling arrays [18]. CEL files were processed using the AffyTiling package in R and returned quantile normalized M values (Figure 2a). A simulated dataset of 40 arrays was then generated using the ‘rnorm’ function from the stats package in R to create probe-wise distributions with the same mean and variance as the experimentally measured dataset (Figure 2a). This simulation dataset allowed us to evaluate DMR finding algorithms in the context of the probe-wise variation that would be detected in an actual experiment. We limited the simulation dataset to 100,000 probes. We subsequently generated DMRs with effect sizes ranging from 0.1 to 2 fold DNA methylation difference and inserted them randomly into the matrix, retaining the positions of the “hidden DMRs” for follow up analysis. DMRs were generated over variable probe lengths using the ‘rnorm’ function separately for the case and control groups. For the case group, the mean of the distribution per probe was increased by the effect size such that the middle probe in the specified DMR represented the maximum value and the mean effect across probes approximated the simulated effect size. The probe specific variation was retained throughout this process. In total, 1,456 hidden DMRs were generated (Additional file 5: Figure S1). Mouse hippocampal DNA methylation data and simulated data are available on the Gene Expression Omnibus under accession numbers GSE43460 and GSE43462.

#### Algorithm performance

We tested the performance of BioTile against TileMap [24] and CHARM [19] to find the randomly inserted DMRs. Due to various features of other peak finding algorithms mentioned above such as limits on dataset size, data input type, or the appropriateness of statistical outputs to the comparison of interest, only TileMap and CHARM could be properly applied to this analysis. For TileMap, we evaluated the performance of hidden Markov model based data smoothing followed by Unbalanced Mixture Subtraction (UMS) based statistical evaluation. Default values were used for genomic smoothing steps employed by CHARM. A comparison of probe specific weights derived by genomic smoothing in CHARM and through meta-analytical weighting in BioTile is depicted at a representative DMR in Figure 2b. Both CHARM and BioTile were run with 1000 permutations for internal statistical evaluation. As the data matrix represents output from preprocessed microarray data, only peak finding algorithms available for the above platforms were implemented without additional data processing or normalization. The ability of the algorithms to identify the DMRs at various effect sizes, probe lengths, and sample sizes of 5, 10,15, and 20 per group was evaluated. A ‘hidden’ DMR was considered found if it overlapped with the genomic coordinates identified as enriched by each algorithm. For all algorithms, DMRs below an FDR significance threshold of 5% were evaluated.

Area under the receiver operator characteristic curves (AUC) were generated to evaluate the sensitivity as a function of specificity of each algorithm. For BioTile and CHARM, FDR significant DMRs overlapping with hidden DMRs represented true positives (TP), non-significant non-overlapping DMRs were true negatives (TN), significant non-overlapping DMRs were false positives (FP) and non-identified hidden DMRs were false negatives (FN). BioTile generated the highest AUC of 0.92 (95% CI = 0.90-0.93, TP = 844, TN = 9,896, FP = 36, FN = 167) while CHARM generated an AUC of 0.74 (95% CI: 0.72-0.75, TP = 625, TN = 12, FP = 0, FN = 692). Because TileMap does not return a list of non-significant DMRs, we could not evaluate the AUC in the same manner. To overcome this, we evaluated each of the 100,000 probes in the simulated dataset for overlap with the significant DMRs per algorithm. The results for BioTile (AUC = 0.95, 95% CI: 0.93-0.95, TP = 16,424, TN = 80,460, FP = 1,686, FN = 1,430) and CHARM (AUC = 0.75, 95% CI:0.73-0.75. TP = 9,015, TN = 81,678, FP = 468, FN = 8,839) were consistent with the previous analysis, while TileMap (AUC = 0.76, 95% CI: 0.73-0.77, TP = 9,462, TN = 82,139, FP = 7, FN = 8,392) performed similarly to CHARM (Additional file 5: Figure S2). The cumulative results suggest that BioTile out performs TileMap and CHARM at the identification of hidden DMRs.

**Figure 3:**
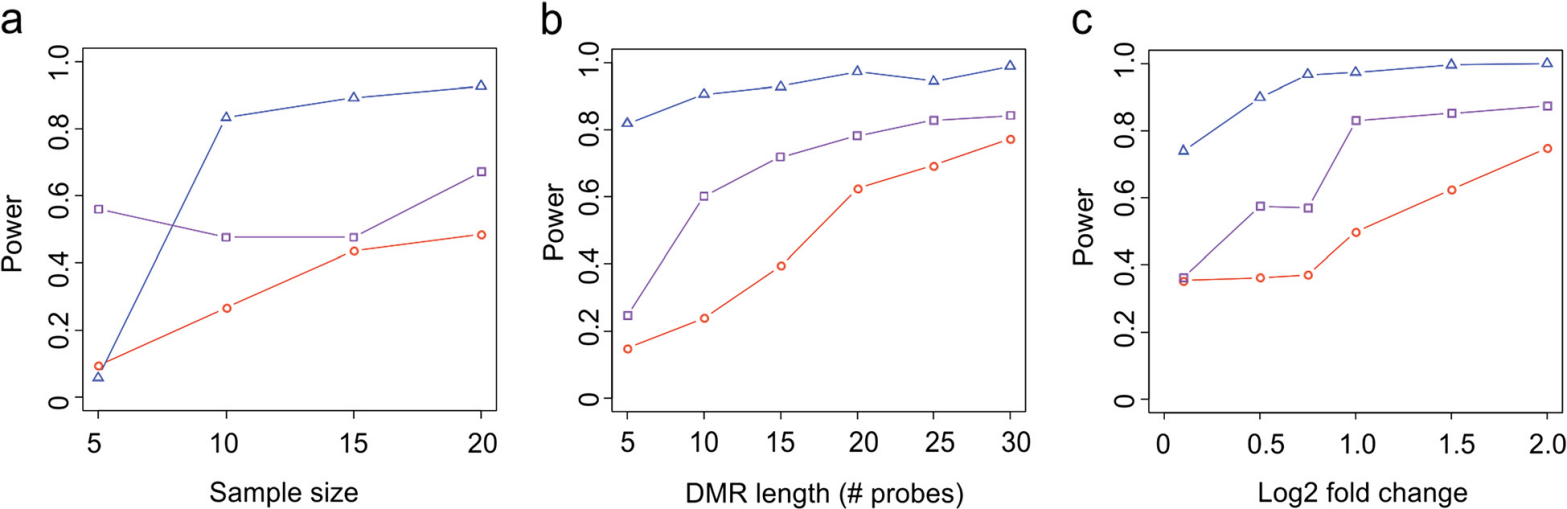
**Algorithm performance.** Plots of the proportion of hidden DMRs identified (power) by BioTile (blue triangles), TileMap (purple squares), and CHARM (red circles) as a function of sample size (**a**), DMR length (**b**), and log_2_ fold change (**c**).

BioTile exhibited a higher power to detect DMRs when evaluating sample sizes of ten cases and controls per group and above; however, at five cases and controls, BioTile did exhibit 82% power when using a nominal as opposed to FDR significance threshold of 5%. At five cases and controls, TileMap significantly out performed both BioTile and CHARM; however, the observed power did not increase significantly with additional sample numbers for this algorithm (Table 1, Figure 3a). At ten samples per group and above, BioTile exhibited significantly higher power to detect DMRs than TileMap and CHARM. For the subsequent evaluation of DMR length and effect size, we held the sample size constant at 20 samples per group.

**Table 1:** Algorithm power

**Sample size**	**BioTile**	**TileMap**	**CHARM**	**BioTile vs. TileMap**	**BioTile vs. CHARM**
5	0.06	0.56	0.09	OR = 0.05, p = 4.6 × 10^-182^	OR = 0.60, p = 1.2 × 10^-03^
10	0.83	0.48	0.27	OR = 5.45, p = 5.1 × 10^-81^	OR = 14.66, p = 1.1 × 10^-196^
15	0.89	0.48	0.44	OR = 8.34, p = 1.7 × 10^-112^	OR = 9.83, p = 5.9 × 10^-132^
20	0.93	0.67	0.49	OR = 5.17, p = 7.7 × 10^-52^	OR = 11.70, p = 4.2 × 10^-133^
**DMR length (# probes)**					
5	0.82	0.25	0.15	OR = 11.39, p = 8.9 × 10^-29^	OR = 20.14, p = 7.2 × 10^-40^
10	0.91	0.60	0.24	OR = 3.64, p = 1.1 × 10^-07^	OR = 19.92, p = 2.6 × 10^-39^
15	0.93	0.72	0.39	OR = 4.86, p = 1.0 × 10^-08^	OR = 17.82, p = 2.6 × 10^-32^
20	0.97	0.78	0.62	OR = 9.25, p = 2.4 × 10^-09^	OR = 22.21, p = 2.4 × 10^-22^
25	0.95	0.83	0.69	OR = 3.65, p = 1.2 × 10^-04^	OR = 7.12, p = 3.5 × 10^-11^
30	0.99	0.84	0.77	OR = 18.65, p = 2.2 × 10^-08^	OR = 29.94, p = 2.9 × 10^-13^
**Log**_**2 **_**fold change**					
0.1	0.74	0.36	0.35	OR = 4.93, p = 7.7 × 10^-19^	OR = 5.18, p = 7.0 × 10^-20^
0.5	0.90	0.58	0.36	OR = 7.26, p = 1.1 × 10^-14^	OR = 18.17, p = 1.8 × 10^-33^
0.75	0.97	0.57	0.37	OR = 20.17, p = 1.1 × 10^-10^	OR = 41.03, p = 2.4 × 10^-18^
1	0.97	0.83	0.50	OR = 7.90, p = 5.0 × 10^-08^	OR = 43.53, p = 3.5 × 10^-41^
1.5	1.00	0.85	0.63	OR = 55.96, p = 2.9 × 10^-14^	OR = 194.39, p = 1.9 × 10^-41^
2	1.00	0.88	0.75	OR = Inf, p = 1.4 × 10^-03^	OR = Inf, p = 4.0 × 10^-07^

A table depicting the power of each algorithm to identify ‘hidden’ DMRs inserted into the simulated data matrix. Fisher’s odds ratios over 1 denote a higher proportion of DMRs identified by BioTile relative to TileMap and CHARM, respectively.

BioTile was the only algorithm to exhibit greater than 80% power to identify small DMRs (five probes). At all probe lengths evaluated, BioTile exhibited significantly higher power relative to the other algorithms (Table 1). All three algorithms demonstrated a consistent increase in power with increasing DMR length (Figure 3b).

Small effect size DMRs may be expected in heterogeneous tissues such as brain and blood, where the epigenetic contribution of phenotype irrelevant cell types may dilute the observable effect size. At the lowest fold change of 0.1, BioTile identified 74% of DMRs, significantly more than that identified by the other algorithms (CHARM = 35%, Fisher’s Exact OR = 5.2, p = 7 × 10^-20^, TileMap = 36%, Fisher’s Exact OR = 4.9, p =7.7 × 10^-19^) (Table 1, Figure 3c).

**Figure 4:**
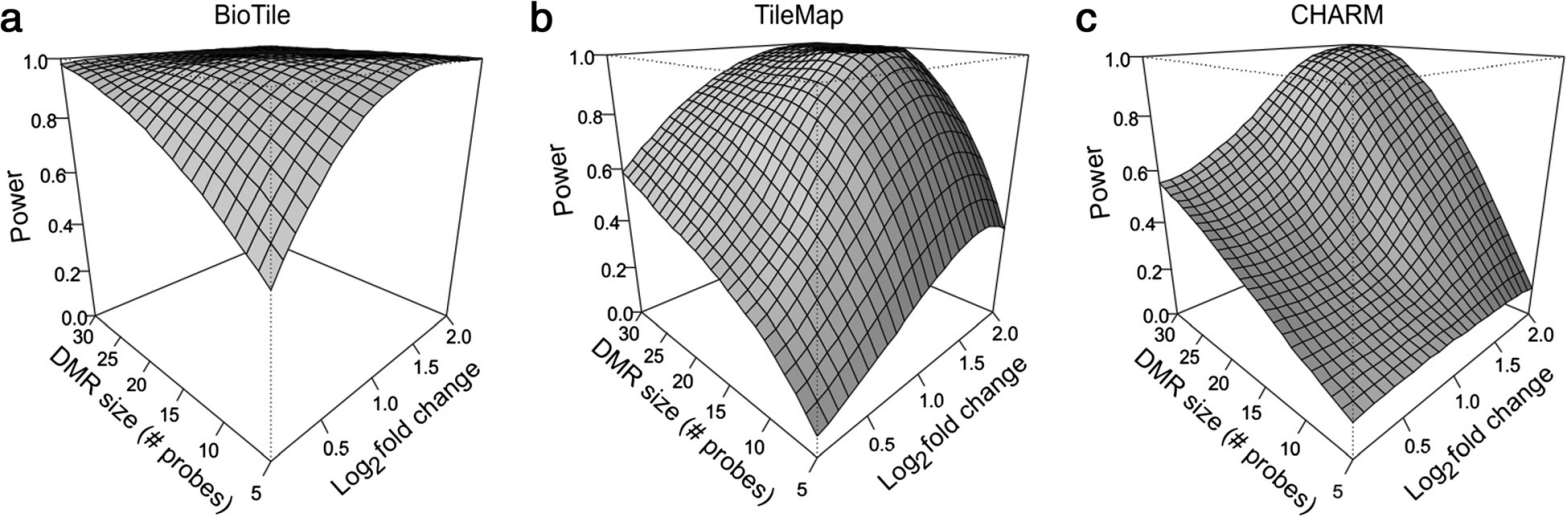
**Interaction of DMR characteristics on algorithm performance.** Three dimensional depictions of non-parametric loess models fitting power (y-axis) as a function of DMR size (x-axis) and effect size (z-axis) for BioTile (**a**), TileMap (**b**), and CHARM (**c**).

Importantly, these power calculations reflect the total proportion of hidden DMRs detected; however, the power of each algorithm to identify a range of DMRs is a function of both DMR length and effect size (Figure 4). The higher power of BioTile to identify DMRs across a range of smaller effect sizes and DMR lengths suggests it represents an ideal tool to employ prior to performing pathway analyses, as all implicated DMRs are likely to represent the true epigenomic landscape of changes in response to an independent variable of interest as opposed to representing only the largest and longest of changes.

#### Comparison of algorithms in biological datasets

We evaluated the performance of BioTile, TileMap, and CHARM at identifying genomic regions exhibiting differential epigenetic changes in response to early life environment in three datasets. The first two datasets derive from a study by McGowan et al., and use custom Agilent tiling microarrays to measure both chromatin immunoprecipitation enriched histone3 lysine9 tri-methylation (H3K9me3) marks and Methylated DNA Immunoprecipitation (MeDIP) enriched DNA methylation from rats born to high or low licking and grooming mothers [28]. The third dataset was generated by Suderman et al., using custom human Agilent microarrays to investigate MeDIP enriched DNA methylation associations to early life trauma [29]. We selected these studies as they are performed both on tiling microarrays and in the hippocampus, a brain region heavily implicated in psychiatric phenotype and consistent with that from which the simulated dataset was modeled. Seven regions were validated in the H3K9me3 study while both MeDIP datasets validated eleven DNA methylation regions. The performance of each algorithm to find these ‘true differences’ and classify them as significant is depicted in Table 2 and appears relatively consistent with the projected power as a function of the sample size for each dataset (Figure 3a). These findings corroborate the simulated results above and demonstrate that BioTile has the highest power to detect true DERs.

**Table 2:** Algorithm performance in biological datasets

**Dataset**	**Interrogated mark**	**# group 1**	**# group 2**	**BioTile**	**TileMap**	**CHARM**
					**# DERs returned**	
1	H3K9me3	12	9	5,315	62	83
2	DNA methylation	6	9	5,767	27	105
3	DNA methylation	10	9	5,558	821	100
					**Proportion of validated DERs found**	
1	H3K9me3	12	9	100%	28%	0%
2	DNA methylation	6	9	100%	9%	0%
3	DNA methylation	10	9	100%	9%	0%
					**Proportion of validated DERs significant**	
1	H3K9me3	12	9	71%	28%	0%
2	DNA methylation	6	9	45%	9%	0%
3	DNA methylation	10	9	55%	9%	0%

A table depicting algorithm performance when applied to datasets derived from the McGowan et al., and Suderman et al., studies investigating the effects of early life environmental influence on epigenetic marks in the hippocampus.

## Conclusion

The BioTile Perl application represents a simple and effective means to identify DERs in genome-scale data. BioTile out performs a number of comparable algorithms designed for the analysis of ChIP-chip data and is not confined to the analysis of a single tiling array platform. Future iterations of the algorithm may focus on the analysis of next-generation sequencing data.

Running the BioTile Perl script is simple and requires only a properly formatted data matrix file and an annotation file containing the comparison and any covariates of interest. The simplicity of BioTile is designed to increase the utility of this bioinformatics resource to the general scientific community.

## Availability and requirements

**Project name:** BioTile

**Project home page:**http://psychiatry.igm.jhmi.edu/kaminsky/software.htm

**Operating systems:** Platform independent

**Programming language:** Perl

**License:** None

## Competing interests

The authors declare no competing interests.

## Authors’ contributions

All authors contributed significantly to the final version of the manuscript. JG performed DNA methylation experiments, MA, KT, and TG generated the experimental animals, and ZK conceptualized and wrote the algorithm and manuscript. All authors read and approved the content.

## Supplementary Material

Additional file 1 A perl script containing the BioTile algorithm. Click here for file

Additional file 2 An example dataset containing the first 5000 data points from 5 randomly selected case and control mice from the simulated data matrix. Click here for file

Additional file 3 **An example annotation file required to run BioTile on the example dataset available in Additional File **2**.** Click here for file

Additional file 4 A readme file outlining basic instructions for running BioTile. A more detailed web based vignette is available on the project homepage. Click here for file

Additional file 5: Figure S1 Simulated data distributions. The distributions of mean DNA methylation log2 fold change of 20 case vs. 20 control microarrays are depicted for the distribution devoid of inserted DMRS (Null distribution) and for the hidden DMRs. **Figure S2**. Receiver operator characteristic curves. Receiver operator characteristic curves are plotted depicting the sensitivity (y-axis) as a function of the specificity (x-axis) to identify hidden DMRs for BioTile (a), TileMap (b), and CHARM (c). Click here for file
