# Supplementary material for: BioTile, A Perl based tool for the identification of differentially enriched regions in tiling microarray data
Source: BMC Bioinformatics. 2013 Mar 3;14:76. doi: 10.1186/1471-2105-14-76 (PMC3599767; doi:10.1186/1471-2105-14-76)
Supplement: Additional file 5: Figure S1 — Simulated data distributions. The distributions of mean DNA methylation log2 fold change of 20 case vs. 20 control microarrays are depicted for the distribution devoid of inserted DMRS (Null distribution) and for the hidden DMRs. Figure S2. Receiver operator characteristic curves. Receiver operator characteristic curves are plotted depicting the sensitivity (y-axis) as a function of the specificity (x-axis) to identify hidden DMRs for BioTile (a), TileMap (b), and CHARM (c). [file 1471-2105-14-76-S5.docx]

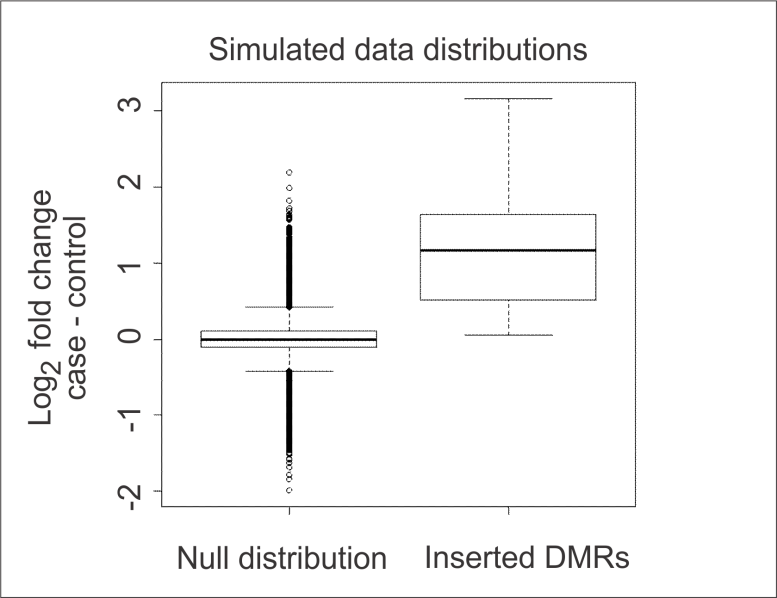


**Supplementary Figure 1.** **Simulated data distributions**

The distributions of mean DNA methylation log_2_ fold change of 20 case vs. 20 control microarrays are depicted for the distribution devoid of inserted DMRS (Null distribution) and for the hidden DMRs.


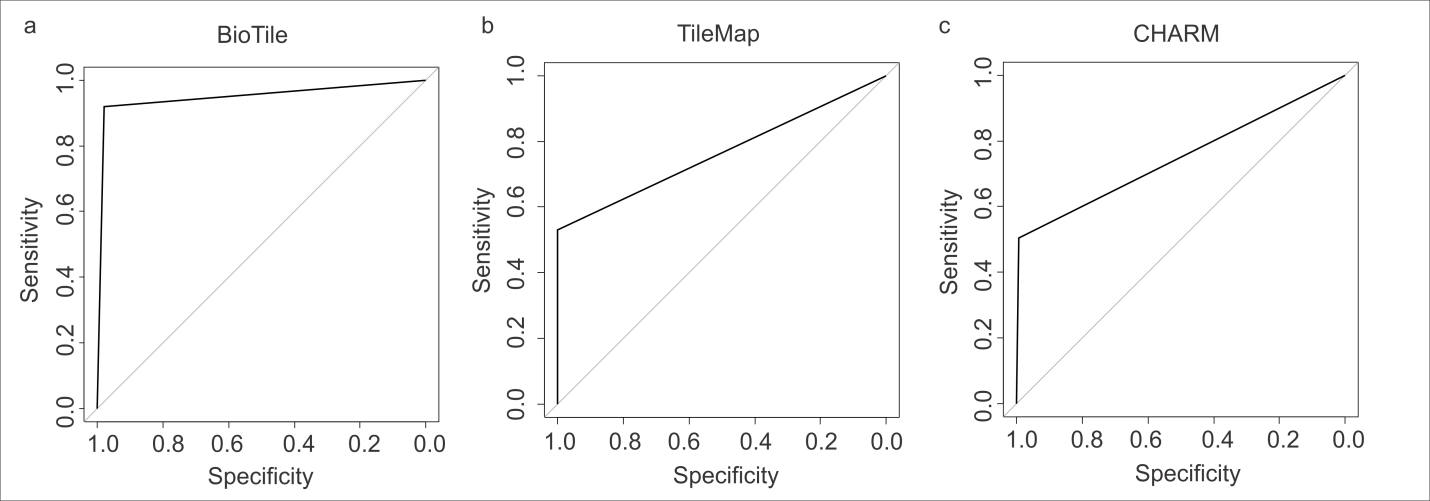


**Supplementary Figure 2.** **Receiver operator characteristic curves**

Receiver operator characteristic curves are plotted depicting the sensitivity (y-axis) as a function of the specificity (x-axis) to identify hidden DMRs for BioTile (a), TileMap (b), and CHARM (c).
